# Molecular Mechanisms Underlying Atherosclerosis and Current Advances in Targeted Therapeutics

**DOI:** 10.3390/ijms27020634

**Published:** 2026-01-08

**Authors:** Bo Zhu

**Affiliations:** Vascular Biology Program, Boston Children’s Hospital, Department of Surgery, Harvard Medical School, Boston, MA 02115, USA; bo.zhu@childrens.harvard.edu

**Keywords:** atherosclerosis, endothelial dysfunction, inflammation, foam cells, non-coding RNA, smooth muscle cells, lipid metabolism

## Abstract

Atherosclerosis is a chronic, multifactorial vascular disease and the leading global cause of cardiovascular morbidity. Its development reflects interconnected disturbances in lipid metabolism, endothelial function, inflammation, smooth muscle cell (SMC) phenotypic switching, and extracellular matrix remodeling. Genetic predisposition, including monogenic disorders such as familial hypercholesterolemia and polygenic risk variants, modulates disease susceptibility by altering lipid homeostasis as well as inflammatory and thrombotic pathways. Epigenetic regulators and noncoding RNAs, such as histone modifications, microRNAs, and long noncoding RNAs, further shape gene expression and link environmental cues to vascular pathology. Endothelial injury promotes lipoprotein retention and oxidation, triggering monocyte recruitment and macrophage-driven foam cell formation, cytokine secretion, and necrotic core development. Persistent inflammation, macrophage heterogeneity, and SMC plasticity collectively drive plaque growth and destabilization. Emerging insights into immune cell metabolism, intracellular signaling networks, and novel regulatory RNAs are expanding therapeutic possibilities beyond lipid-lowering. Current and evolving treatments include statins, proprotein convertase subtilisin/kexin type 9 (PCSK9) inhibitors, anti-inflammatory agents targeting interleukin-1 beta (IL-1β) or NOD-, LRR-, and pyrin domain-containing protein 3 (NLRP3), and advanced approaches such as gene editing, siRNA, and nanoparticle-based delivery. Integrating multi-omics, biomarker-guided therapy, and precision medicine promises improved risk stratification and next-generation targeted interventions. This review summarizes recent molecular advances and highlights translational opportunities for enhancing atherosclerosis prevention and treatment.

## 1. Introduction

**Figure 1:**
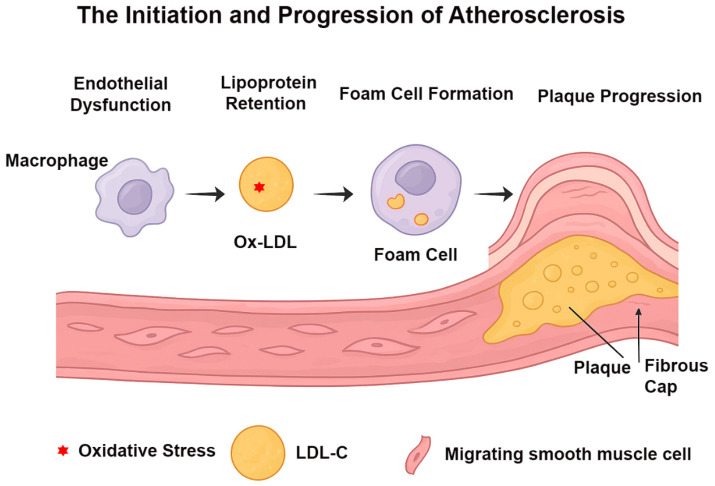
Endothelial dysfunction facilitates subendothelial retention of LDL-C, which subsequently undergoes oxidative modification to form oxidized LDL (Ox-LDL). Recruited macrophages internalize Ox-LDL and differentiate into foam cells, promoting early lesion development. Progressive accumulation of foam cells and persistent inflammatory activation drive plaque expansion. Migrating smooth muscle cells contribute to extracellular matrix deposition and formation of a fibrous cap over the lipid-rich core, culminating in mature atherosclerotic plaque formation (Created with BioRender.com).

Atherosclerosis is the primary driver of cardiovascular disease (CVD), which remains a major cause of illness and death worldwide [1,2]. The development of atherosclerosis is a multifaceted process involving disruptions in lipid balance, persistent inflammation of blood vessels, and dysfunction of cells within the arterial walls [3,4,5] (Figure 1). Key traditional risk factors, such as high levels of low-density lipoprotein cholesterol (LDL-C), metabolic syndrome, hypertension, diabetes, and tobacco use, play a significant role in promoting the disease [6,7]. Gaining a deep understanding of the cellular and molecular pathways underlying atherogenesis is essential for the development of innovative treatment strategies.

**Figure 2:**
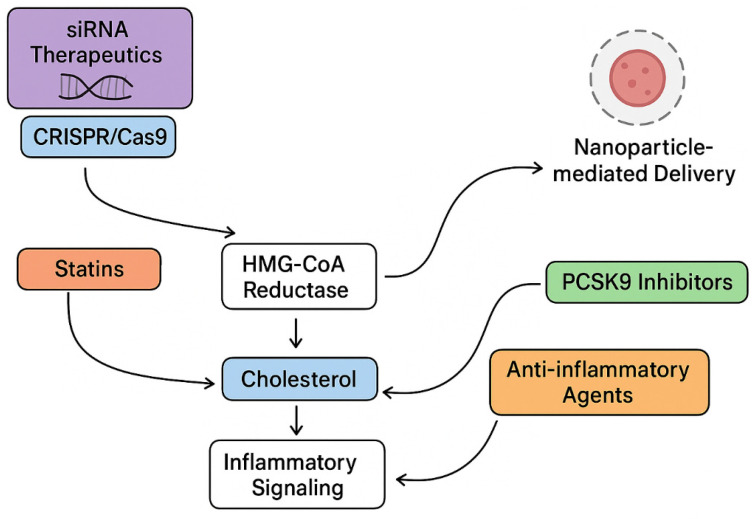
The schematic highlights major molecular targets and regulatory pathways involved in controlling cholesterol homeostasis and inflammatory signaling. Anti-inflammatory agents modulate upstream inflammatory cascades that influence lipid metabolism. Statins inhibit 3-hydroxy-3-methylglutaryl-coenzyme A (HMG-CoA) reductase, thereby suppressing endogenous cholesterol synthesis, while PCSK9 inhibitors enhance low-density lipoprotein (LDL) clearance by increasing LDL receptor availability. Nucleic acid-based therapeutics, including small interfering RNA (siRNA) and CRISPR/Cas9 genome-editing platforms, modulate the expression of genes implicated in cholesterol regulation. Nanoparticle-based delivery systems facilitate the targeted transport of these therapeutic modalities. Together, these interventions converge on interconnected pathways governing cholesterol levels and the associated inflammatory response (Created with BioRender.com).

**Figure 3:**
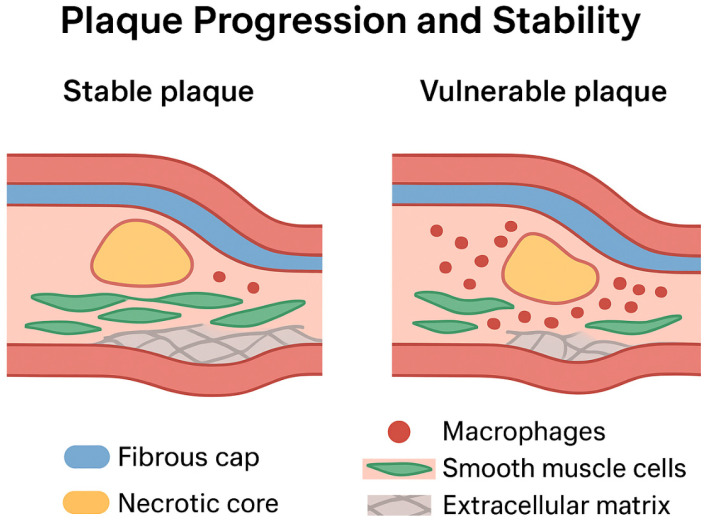
Structural characteristics distinguishing stable and vulnerable atherosclerotic plaques. The illustration contrasts the morphological features of a stable plaque (**left**) with those of a vulnerable plaque (**right**). Stable plaques typically display a thick, collagen-rich fibrous cap, abundant and well-organized smooth muscle cells, limited macrophage infiltration, and a relatively small necrotic core—features associated with reduced propensity for rupture. In contrast, vulnerable plaques exhibit a thin and weakened fibrous cap, an expanded lipid-rich necrotic core, extensive macrophage accumulation, and marked extracellular matrix degradation. These structural alterations promote heightened inflammation, cap destabilization, and an increased likelihood of plaque rupture and thrombosis. Color coding delineates major cellular and structural components, including the fibrous cap, necrotic core, macrophages, smooth muscle cells, and extracellular matrix (Created with BioRender.com).

Over recent decades, pharmacological interventions such as statins and PCSK9 inhibitors have successfully reduced cardiovascular events by lowering circulating atherogenic lipoproteins and modulating lipid-driven vascular injury [8,9] (Figure 2). Despite these advances, substantial residual risk remains, particularly due to non-lipid factors, including persistent vascular inflammation, immune cell activation, and plaque instability [8,10]. These observations highlight the necessity of investigating molecular processes underlying endothelial dysfunction, lipoprotein retention and modification, immune cell recruitment and activation, smooth muscle cell phenotypic remodeling, and extracellular matrix remodeling that collectively contribute to plaque progression and vulnerability [11,12,13] (Figure 3).

Emerging research has identified previously underappreciated molecular regulators of atherosclerosis. These include endocytic and scavenger receptor pathways, noncoding RNAs that modulate gene expression, metabolic reprogramming within immune cells, and novel signaling cascades that influence lesion development at multiple stages [14,15,16]. Insights into these molecular mechanisms not only deepen our understanding of disease pathophysiology but also provide a foundation for next-generation, targeted therapies, such as cell-specific drug delivery, RNA-based interventions, gene editing, and immunomodulation [17,18].

In this review, we synthesize recent advances in molecular insights into atherosclerosis, tracing the continuum from early endothelial alterations to advanced plaque formation. We further examine how these mechanistic insights inform current therapies and emerging strategies, aiming to provide a comprehensive, up-to-date resource that links basic molecular research with translational and clinical applications.

## 2. Genetic Predisposition of Atherosclerosis

**Figure 4:**
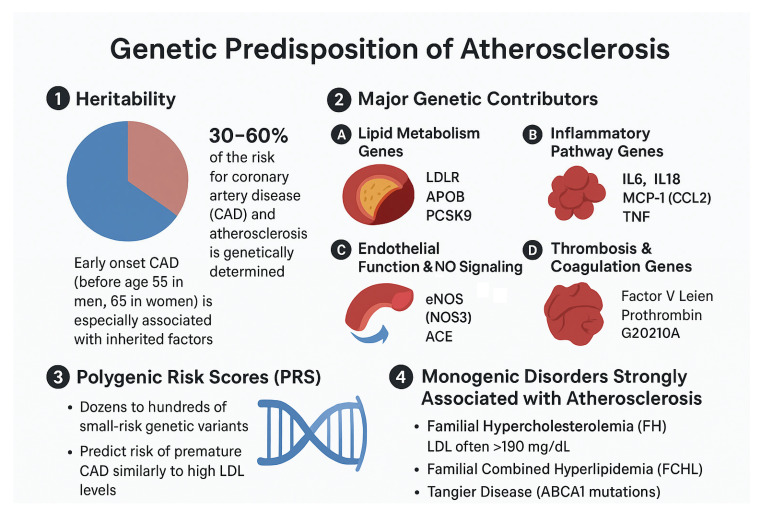
This figure summarizes key genetic determinants contributing to interindividual susceptibility to atherosclerosis. (**1**) Heritability: An estimated 30–60% of the risk for coronary artery disease (CAD) and atherosclerosis is genetically determined. Familial clustering is particularly strong in early-onset CAD (before age 55 in men and 65 in women). (**2**) Major Genetic Contributors: Several classes of genes influence atherosclerotic initiation and progression, including: (**A**) Lipid metabolism genes (e.g., LDLR, APOB, PCSK9) that regulate low-density lipoprotein (LDL) clearance; (**B**) Inflammatory pathway genes (e.g., IL6, IL18, MCP-1/CCL2, TNF) that modulate vascular inflammation; (**C**) Endothelial function and nitric oxide (NO) signaling genes, such as eNOS/NOS3 and ACE, which regulate vasomotor tone and oxidative stress; and (**D**) Thrombosis and coagulation genes (e.g., Factor V Leiden, Prothrombin G20210A) that influence prothrombotic propensity. (**3**) Polygenic Risk Scores (PRS): Aggregated combinations of numerous small-effect genetic variants collectively shape CAD risk and can predict premature disease with discrimination comparable to elevated LDL cholesterol. (**4**) Monogenic Disorders Associated with Accelerated Atherosclerosis: Conditions such as familial hypercholesterolemia (FH; caused by mutations in LDLR, APOB, or PCSK9), familial combined hyperlipidemia (FCHL), and Tangier disease (ABCA1 mutations) result in marked dyslipidemia and substantially increased atherosclerotic burden (Created with BioRender.com).

Atherosclerosis is a chronic and progressive vascular condition marked by the buildup of lipids, inflammatory cells, and fibrous tissue within the arterial wall. Although lifestyle and environmental factors such as dietary habits, smoking, and sedentary behavior play significant roles, a considerable portion of individual susceptibility is governed by genetic factors. Heritability analyses indicate that genetic influences account for approximately 30–60% of the risk for coronary artery disease (CAD) and related atherosclerotic disorders, particularly in cases with early onset [19,20]. These genetic determinants affect diverse biological processes, including lipid metabolism, endothelial integrity, inflammatory pathways, and thrombosis (Figure 4), collectively modulating an individual’s inherent predisposition to plaque formation and disease progression.

Genetic influences on lipid metabolism account for a significant portion of inherited susceptibility to atherosclerosis. Variants in genes that regulate low-density lipoprotein (LDL) levels, such as LDL receptor (LDLR), apolipoprotein B (APOB), and proprotein convertase subtilisin/kexin type 9 (PCSK9), can markedly elevate LDL concentrations, as observed in familial hypercholesterolemia, a monogenic disorder associated with early and aggressive plaque formation [21,22,23]. In addition, genes involved in high-density lipoprotein (HDL) production and triglyceride clearance, including apolipoprotein A-I (APOA1), cholesteryl ester transfer protein (CETP), and lipoprotein lipase (LPL), shape lipoprotein composition and influence the balance between lipid accumulation and removal within the arterial wall [24,25,26]. Disruptions in these pathways lead to increased levels of atherogenic lipoproteins, greater LDL retention in the intima, and enhanced lipid oxidation, collectively promoting the initiation and early progression of atherosclerotic lesions.

Beyond lipid regulation, genetic determinants of vascular inflammation significantly modulate atherosclerotic susceptibility. Polymorphisms in cytokine and chemokine genes, such as interleukin 6 (IL6), interleukin 1 beta (IL1β), tumor necrosis factor alpha (TNFα), and C-C motif chemokine ligand 2 (CCL2), influence the magnitude and persistence of the inflammatory response within the arterial microenvironment [27,28,29]. These variants modulate endothelial activation, monocyte recruitment, and macrophage polarization, thereby influencing both the initiation of fatty streaks and the transition to complex, inflamed lesions. Similarly, genetic variation in endothelial nitric oxide synthase (NOS3) and components of the renin–angiotensin system, including the angiotensin-converting enzyme (ACE) insertion/deletion polymorphism, impair nitric oxide bioavailability, promote oxidative stress, and facilitate endothelial dysfunction—an early and critical event in atherogenesis [30,31,32].

The genetic architecture of atherosclerosis also encompasses factors that affect coagulation and thrombosis, which influence the clinical complications of plaque rupture. Variants such as Factor V Leiden, prothrombin G20210A, and polymorphisms in PAI-1 modulate thrombin generation, fibrinolysis, and clot stability [33,34,35]. While these genes do not directly initiate plaque formation, they determine the likelihood of thrombotic events, thereby shaping overall cardiovascular risk. In most individuals, however, susceptibility arises not from single high-impact variants but from the cumulative effect of numerous small-effect alleles. Polygenic risk scores integrating thousands of loci have demonstrated strong predictive value for coronary artery disease and can identify individuals who benefit most from early preventive interventions, including lipid-lowering therapy [36,37,38].

Importantly, the interplay between genetic predisposition and environmental exposures shapes the trajectory of atherosclerosis. Lifestyle factors such as smoking, hypertension, hyperglycemia, and dietary patterns can amplify or unmask underlying genetic susceptibility through mechanisms involving oxidative stress, inflammation, and epigenetic modifications [39,40,41]. Epigenetic regulation, including DNA methylation and histone remodeling, provides an additional layer of control linking environmental stimuli to altered gene expression profiles in vascular tissues, thereby influencing disease manifestation. Consequently, the phenotypic expression of genetic risk is highly context-dependent and can vary substantially among individuals with similar genotypes.

In summary, genetic predisposition to atherosclerosis reflects a complex interplay between monogenic disorders, polygenic variation, and environmentally modulated gene expression. Advances in genomics, transcriptomics, and epigenetics have provided critical insights into the molecular pathways underlying inherited risk, enabling refined risk stratification and the development of personalized therapeutic strategies. Ongoing integration of genetic, molecular, and environmental data is expected to enhance the prevention and treatment of atherosclerotic cardiovascular disease.

## 3. Molecular Mechanisms of Atherosclerosis

**Figure 5:**
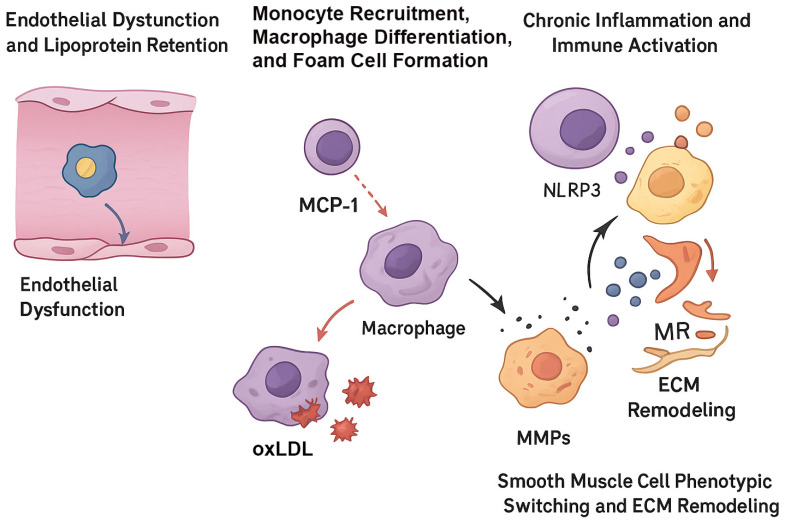
Major cellular and molecular events driving atherogenesis. This schematic depicts the key steps involved in the initiation and progression of atherosclerosis. Endothelial dysfunction facilitates the subendothelial retention of atherogenic lipoproteins and promotes the recruitment of circulating monocytes. Upon entry into the intima, monocytes differentiate into macrophages, which internalize oxidized low-density lipoprotein (oxLDL) and subsequently transform into foam cells. Foam cells secrete matrix metalloproteinases and pro-inflammatory mediators that drive smooth muscle cell phenotypic switching, extracellular matrix remodeling, and sustained inflammation within the arterial wall. Abbreviations: MCP-1, monocyte chemoattractant protein-1; ECM, extracellular matrix; MMPs, matrix metalloproteinases (Created with BioRender.com).

Atherosclerosis is not merely a lipid storage disease, but rather a chronic, dynamic interplay of lipid metabolism, vascular cell biology, and immune-mediated inflammation. The following subsections outline key molecular and cellular mechanisms that drive atherogenesis and plaque progression (Figure 5).

### 3.1. Endothelial Dysfunction and Lipoprotein Retention

The process of atherosclerosis often begins with impairment of the vascular endothelium, the innermost cell layer that lines blood vessels. Under physiological conditions, endothelial cells maintain vascular homeostasis by regulating vascular tone, leukocyte adhesion, and controlling lipoprotein transport [42]. However, exposure to cardiovascular risk factors, such as disturbed flow, hyperlipidemia, or smoking, compromises endothelial integrity [42]. Endothelial dysfunction increases vascular permeability, alters the balance of vasoactive mediators, including reduced nitric oxide production, increased reactive oxygen species, and promotes a pro-inflammatory, pro-adhesive state [5,43,44].

In this dysfunctional environment, circulating apoB-containing lipoproteins (chiefly LDL, but also VLDL or remnants) penetrate the endothelial barrier and accumulate in the subendothelial “intima” layer [4,45]. Once trapped, these lipoproteins, especially LDL, are susceptible to chemical modifications, prominently oxidation, leading to the formation of oxidized LDL [46,47,48]. OxLDL is highly atherogenic; it triggers endothelial activation, upregulates expression of adhesion molecules such as vascular cell adhesion molecule 1 (VCAM-1), intercellular adhesion molecule 1 (ICAM-1), and selectins, which facilitate the adherence and transmigration of circulating monocytes into the intima [10,49,50].

Furthermore, oxLDL amplifies oxidative stress and inflammatory signaling within the endothelium, promoting the secretion of chemokines and cytokines that recruit additional immune cells and perpetuate local inflammation [51,52]. This feed-forward cycle enhances further lipoprotein oxidation, endothelial impairment, and immune cell accumulation.

Thus, endothelial dysfunction and lipoprotein retention create a permissive milieu for atherogenesis. These early events, including endothelial activation, lipid accumulation, and immune cell recruitment, set the stage for the progression of atherosclerotic lesions, bridging the transition from a healthy vascular wall to an atheroprone state [13,15].

### 3.2. Monocyte Recruitment, Macrophage Differentiation, and Foam Cell Formation

Following endothelial activation, circulating monocytes adhere to the vascular endothelium through interactions with upregulated adhesion molecules [53]. Chemokines, particularly monocyte chemoattractant protein-1 (MCP-1), and growth factors such as macrophage colony-stimulating factor (M-CSF) orchestrate their directed transmigration into the subendothelial intima, where they differentiate into tissue macrophages [4,49,50]. This recruitment process is tightly regulated, involving gradients of chemotactic signals and localized endothelial cues that ensure selective infiltration at atheroprone sites.

Within the intima, macrophages acquire a pro-atherogenic phenotype characterized by the expression of diverse scavenger receptors (SRs), including Cluster of Differentiation 36 (CD36) and lectin-like oxidized low-density lipoprotein receptor-1 (LOX-1). These receptors specifically recognize the internalized oxidized LDL, mediating its uptake in a manner independent of intracellular cholesterol content [49,54,55,56,57,58]. Unlike the classical LDL receptor, which is downregulated when intracellular cholesterol levels rise, scavenger receptor-mediated uptake persists unchecked, leading to massive lipid accumulation within the macrophage cytoplasm. This process drives the transformation of macrophages into lipid-laden “foam cells,” which constitute the cellular core of the early atherosclerotic lesions, also referred to as fatty streaks [13,59,60].

Foam cells are metabolically active entities, not passive lipid reservoirs. They secrete a spectrum of pro-inflammatory cytokines, including tumor necrosis factor-alpha (TNF-α), IL-1β, and interleukin-6 (IL-6), thereby sustaining local inflammation, oxidative stress, and recruitment of additional immune cells to the lesion site [10,49,51]. In advanced lesions, both macrophage- and smooth muscle cell-derived foam cells may undergo apoptosis or other forms of regulated cell death. If efferocytosis, the clearance of dead or dying cells, is inefficient, cellular debris accumulates, contributing to the formation of a lipid-rich necrotic core [61,62].

Collectively, monocyte recruitment, macrophage differentiation, and foam cell formation establish a self-perpetuating cycle of lipid accumulation and inflammation. These processes not only drive the expansion of early atherosclerotic lesions but also lay the foundation for plaque progression and complexity, ultimately linking early endothelial dysfunction to advanced, clinically significant atherosclerosis [13,14,15].

### 3.3. Inflammation, Immune Activation, and Chronic Lesion Progression

Atherosclerosis is increasingly recognized not merely as a lipid storage disorder but as a chronic, immune-mediated inflammatory disease of the arterial wall [13,15,51]. Within the evolving lesion microenvironment, foam cells, endothelial cells, and other vascular-resident cells produce a broad spectrum of pro-inflammatory cytokines and chemokines, including interleukin-1 (IL-1), interleukin-6, tumor necrosis factor-alpha, and monocyte chemoattractant protein-1, which collectively enhance recruitment of circulating monocytes, T cells, and other immune effectors [10,13,63]. This establishes a self-amplifying loop in which lipid accumulation drives inflammatory activation, which in turn promotes additional immune cell infiltration and further lipid uptake, perpetuating lesion progression.

At the intracellular level, macrophages sense and respond to a variety of danger-associated molecular patterns (DAMPs), including oxidized lipids, cholesterol crystals, oxidative stress, and lysosomal dysfunction. These stimuli activate cytosolic pattern recognition receptors, most notably the NLRP3 inflammasome [64,65,66]. NLRP3 activation triggers caspase-1-mediated cleavage of pro-IL-1β and pro-IL-18, resulting in secretion of these potent pro-inflammatory cytokines and further amplification of endothelial dysfunction and local inflammation [8,64].

Recent advances in single-cell RNA sequencing of human and murine atherosclerotic plaques have revealed significant heterogeneity within plaque-resident macrophages. Beyond the classical M1 (pro-inflammatory) and M2 (anti-inflammatory) classification, macrophage populations encompass multiple specialized subtypes, including inflammatory macrophages, lipid-loaded or resident-like macrophages, proliferating macrophages, interferon-responding macrophages (IFNIC), and TREM2^hi^ macrophages [67,68,69]. The phenotypic plasticity, metabolic reprogramming, and differential survival of these macrophages critically influence plaque composition, determining the balance between lipid-rich necrotic core and fibrous cap integrity, ultimately affecting plaque stability and vulnerability [67,70,71].

In addition, foam cells and other activated immune cells secrete extracellular matrix (ECM)-degrading enzymes, such as matrix metalloproteinases (MMPs), which degrade collagen and other structural components of the fibrous cap. This enzymatic activity thins the cap, undermines structural integrity, and predisposes plaques to rupture, a key event precipitating thrombosis and acute cardiovascular events [72,73,74].

Collectively, chronic inflammation, immune cell heterogeneity, and ECM remodeling orchestrate the progression from early fatty streaks to complex, high-risk atherosclerotic lesions. This interplay of lipid-driven immune activation and structural remodeling underpins both plaque growth and clinical complications.

### 3.4. Smooth Muscle Cells, Extracellular Matrix, Phenotypic Switching

As atherosclerotic lesions progress, vascular smooth muscle cells (VSMCs) migrate from the medial layer of the vessel wall into the intima in response to chemokines, growth factors, and local inflammatory signals [75,76,77]. Upon entering the intima, VSMCs undergo a process known as phenotypic switching, transitioning from a quiescent, contractile phenotype to a synthetic, proliferative state. This phenotypic modulation is characterized by enhanced proliferation, migration, and robust secretion of extracellular matrix (ECM) proteins, particularly collagen, elastin, and proteoglycans, which contribute to intimal thickening and ECM remodeling [75,77,78].

The ECM produced by synthetic VSMCs forms a fibrous cap, a collagen-rich structure that overlays the lipid-laden necrotic core of the atheromatous plaque. This cap initially stabilizes the lesion by physically isolating thrombogenic lipids and cellular debris from circulating blood, thereby reducing the risk of acute thrombotic events [73,79,80]. However, persistent inflammation, oxidative stress, and proteolytic activity, particularly from matrix metalloproteinases secreted by macrophages and other immune cells, can degrade ECM components, thinning the fibrous cap and compromising plaque stability [72,74,80].

VSMC apoptosis, senescence, and impaired ECM production further exacerbate cap weakening, contributing to the transition from stable plaques to vulnerable plaques, which are highly prone to rupture and subsequent thrombosis [75,78]. Importantly, VSMCs also exhibit remarkable plasticity, with the capacity to transdifferentiate into foam cell-like phenotypes or osteochondrogenic-like cells, further influencing plaque composition, calcification, and stability.

Thus, VSMC phenotypic switching and ECM remodeling are central to both the structural integrity of atherosclerotic plaques and the dynamic processes that determine plaque vulnerability, linking cellular behavior to clinical outcomes in atherosclerotic cardiovascular disease [73,77,79].

### 3.5. Epigenetic Modifications Involved in the Pathogenesis of Atherosclerosis

**Figure 6:**
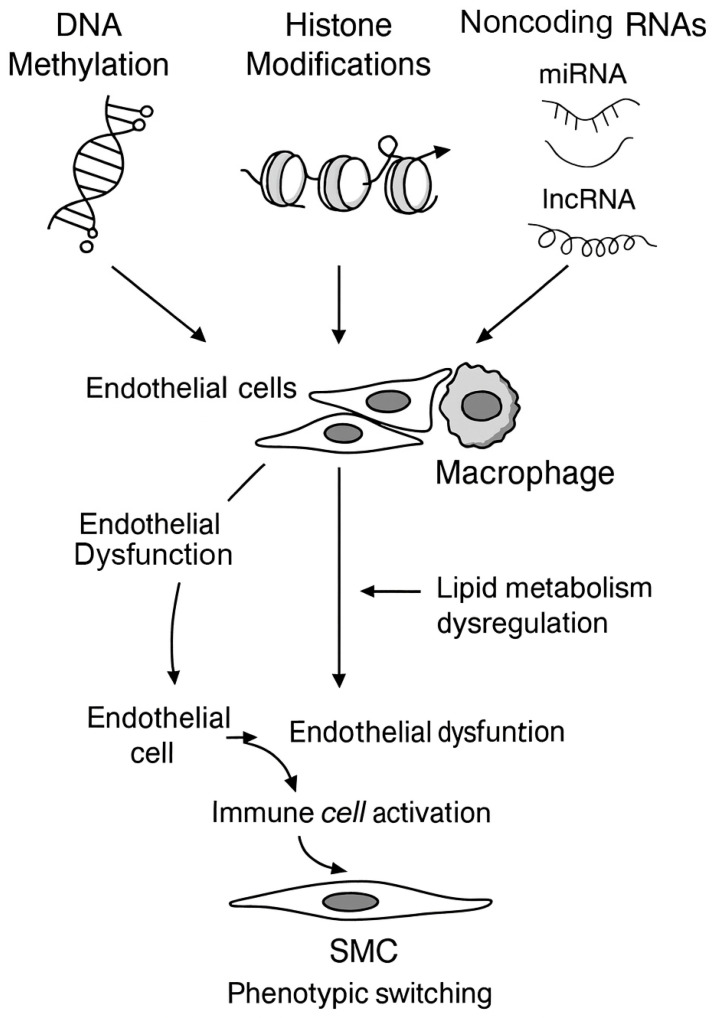
Epigenetic mechanisms in atherosclerosis pathogenesis. DNA methylation, histone modifications, and noncoding RNAs (miRNAs and lncRNAs) regulate critical cellular processes in atherosclerosis. Aberrant epigenetic changes affect endothelial cells, macrophages, and vascular smooth muscle cells (SMCs), resulting in endothelial dysfunction, immune cell activation, dysregulated lipid metabolism, and SMC phenotypic switching. Together, these alterations promote plaque formation, progression, and instability (Created with BioRender.com).

Atherosclerosis is now understood to be shaped not only by genetic predisposition and environmental exposures but also by epigenetic mechanisms that regulate gene activity without altering the underlying DNA sequence. Epigenetic regulators, including DNA methylation, histone modifications, and various noncoding RNAs, play key roles in promoting endothelial dysfunction, modulating immune responses, disturbing lipid metabolic pathways, and driving phenotypic transitions in vascular smooth muscle cells [16,81] (Figure 6).

#### 3.5.1. DNA Methylation

Aberrant DNA methylation patterns are a common feature of atherosclerotic plaques. Widespread hypomethylation in vascular cells can enhance the expression of pro-inflammatory genes, whereas hypermethylation of genes with anti-inflammatory or atheroprotective functions, such as those encoding eNOS or certain ATP-Binding Cassette (ABC) transporters, may accelerate disease development [82,83]. A notable example is the hypermethylation of the ATP-Binding Cassette Transporter A1 (ABCA1) promoter, which diminishes cholesterol efflux from macrophages and promotes their transformation into foam cells [84].

#### 3.5.2. Histone Modifications

Histone post-translational modifications, including acetylation, methylation, and phosphorylation, are crucial determinants of chromatin organization and gene regulation in atherosclerosis. Enzymatic regulators, such as histone acetyltransferases (HATs) and deacetylases (HDACs), influence the expression of inflammatory genes in both endothelial cells and macrophages. Studies have shown that targeting HDAC activity can reduce NF-κB-driven inflammation and limit monocyte infiltration in experimental atherosclerotic models [85]. Additionally, distinct histone methylation marks, including H3K4me3 and H3K27me3, govern macrophage polarization and smooth muscle cell phenotype, thereby affecting plaque composition and stability [86].

#### 3.5.3. Noncoding RNAs

Non-coding RNAs (ncRNAs), including microRNAs (miRNAs) and long non-coding RNAs (lncRNAs), have emerged as essential regulators of the molecular mechanisms underlying atherosclerosis. These regulatory RNAs modulate transcriptional and post-transcriptional programs that influence endothelial homeostasis, lipid metabolism, inflammation, vascular smooth muscle cell (VSMC) phenotype, and plaque stability. Dysregulation of several ncRNAs is directly implicated in atherogenesis, integrating metabolic, environmental, and genetic cues into vascular pathology [16,87,88].

##### MicroRNAs

Endothelial-enriched miR-126 maintains vascular integrity by promoting angiogenic signaling and suppressing leukocyte adhesion, and its reduced expression is closely associated with endothelial injury [89]. In contrast, miR-92a impairs endothelial function by inhibiting KLF2 and KLF4, thereby reducing nitric oxide availability and promoting inflammatory gene expression [90]. miR-21 further modulates endothelial apoptosis and vascular remodeling [91].

Several miRNAs also regulate lipid metabolism. miR-33a/b, hosted within sterol regulatory element-binding transcription factor (SREBF) genes, inhibit ABCA1 and ABCG1, thereby reducing cholesterol efflux and promoting foam-cell formation [92]. miR-122 and miR-148a regulate hepatic lipid synthesis and LDL receptor expression, influencing systemic lipoprotein levels [93,94].

Inflammatory pathways represent another major axis influenced by miRNAs. miR-155 promotes pro-inflammatory macrophage activation [95], whereas miR-146a limits NF-κB signaling and cytokine production, acting as a negative feedback regulator [96]. miR-124 further restricts M1 macrophage polarization [97]. In VSMCs, the miR-145/143 cluster preserves the contractile phenotype, and its downregulation promotes VSMC proliferation and migration [98]. miR-21 and the miR-221/222 cluster similarly drive VSMC proliferation and neointimal expansion [99]. Members of the miR-29 family inhibit extracellular matrix synthesis and may destabilize the fibrous cap [100].

##### Long Non-Coding RNAs

lncRNAs regulate transcriptional, inflammatory, and metabolic pathways in endothelial cells, macrophages, and VSMCs. ANRIL (CDKN2B-AS1), associated with the 9p21 coronary artery disease locus, modulates proliferation, apoptosis, and inflammatory gene networks, linking genomic risk to vascular disease [88]. Several lncRNAs regulate lipid homeostasis, including LeXis, which modulates hepatic cholesterol metabolism via SREBP pathways [101], and MeXis, which enhances macrophage cholesterol efflux by promoting ABCA1 transcriptional activity [102]. MALAT1 influences endothelial proliferation, angiogenesis, and inflammation [103]. Additional inflammatory lncRNAs include LINC00493 and lincRNA-Cox2, which modulate endothelial and macrophage immune activity, respectively [104,105].

lncRNAs also regulate VSMC phenotype and plaque stability. SENCR supports the contractile VSMC phenotype and limits pathological migration [106], whereas SMILR promotes VSMC proliferation and is associated with unstable plaque characteristics [107]. MIAT and lnc-p21 contribute to endothelial dysfunction, apoptosis, and advanced plaque remodeling [108,109].

##### Crosstalk Between MiRNAs and LncRNAs

Interactions between miRNAs and lncRNAs add an additional layer of regulatory control. Many lncRNAs act as competitive endogenous RNAs (ceRNAs), sequestering miRNAs to modulate their downstream targets. For example, CHROME regulates cholesterol efflux pathways through interactions with miR-27b, miR-33a/b, and miR-128 [110]. MALAT1 and ANRIL also modulate inflammatory and proliferative pathways through miRNA sponging [103].

##### Clinical Implications

Dysregulated circulating ncRNAs, including miR-126, miR-155, miR-33, ANRIL, MIAT, and H19, display potential as biomarkers for early atherosclerosis, plaque instability, and cardiovascular risk stratification [111]. Therapeutic strategies targeting ncRNAs, such as anti-miR therapies or lncRNA-directed antisense oligonucleotides, show promise [112,113]. However, challenges related to delivery, specificity, and off-target effects remain. Combining ncRNA-based diagnostics with genomic profiling and lipid-lowering therapies may enable more precise risk assessment and treatment of atherosclerotic disease.

#### 3.5.4. Epigenetic Crosstalk with Metabolic and Inflammatory Pathways

Epigenetic modifications are influenced by environmental and metabolic cues, such as oxidized LDL, hyperglycemia, and hypoxia. For instance, metabolic reprogramming of macrophages can alter histone acetylation patterns, enhancing pro-inflammatory gene expression, while DNA methylation changes in endothelial cells can amplify oxidative stress responses [114,115]. This crosstalk contributes to sustained inflammation, impaired cholesterol handling, and SMC phenotypic switching—hallmarks of atherosclerotic progression.

#### 3.5.5. Therapeutic Implications

Targeting epigenetic regulators presents a promising strategy for atherosclerosis. HDAC inhibitors, DNA methyltransferase inhibitors, and miRNA-based therapeutics have shown efficacy in preclinical models by reducing inflammation, promoting cholesterol efflux, and stabilizing plaques [16,87,116]. Integrating epigenetic therapies with conventional lipid-lowering and anti-inflammatory strategies may offer synergistic benefits for precision cardiovascular medicine.

### 3.6. Emerging Molecular Players and Metabolic Reprogramming

Recent advances highlight additional molecular layers that influence atherosclerosis beyond classical lipid-inflammation paradigms [49,73]. In particular, metabolic reprogramming within immune cells, such as macrophages, is now seen as a determinant of their phenotype, function, and fate within plaques [114,117,118]. For example, shifts toward glycolysis, metabolic reprogramming in macrophages under hypoxic, inflammatory, or lipid-rich plaque microenvironments can promote pro-inflammatory macrophage activation (“M1-like”), increased reactive oxygen species (ROS) production, and enhanced plaque vulnerability [119,120].

Cholesterol handling pathways inside macrophages are also critical. Reverse cholesterol transport, mediated by transporters such as ABCA1, ABCG1, and Scavenger Receptor Class B Type 1 (SR-B1), enables efflux of cholesterol from macrophages to external acceptors such as high-density lipoprotein (HDL), which reduces lipid load and foam cell formation [121,122,123]. Dysregulation of these transporters, e.g., decreased ABCA1/ABCG1 expression, whether by inflammatory signaling, metabolic stress, or epigenetic modulation, leads to impaired cholesterol efflux, lipid accumulation, and foam cell formation [114,124,125].

Moreover, emerging data implicates noncoding RNAs, e.g., microRNAs, in regulating lipid metabolism, cholesterol efflux, inflammatory signaling, and SMC phenotype [16,126]. Also, signaling pathways such as mTOR, Wnt, and others, often sensitive to metabolic and oxidative stress, are involved in controlling immune cell activation, survival, and ECM remodeling [127,128].

Collectively, these molecular layers, metabolic reprogramming, cholesterol-handling transport, noncoding RNA regulation, and intracellular signaling, add complexity to atherogenesis but also offer new therapeutic entry points beyond classical lipid-lowering [14,71,73].

## 4. Therapeutic Advances in Atherosclerosis

**Figure 7:**
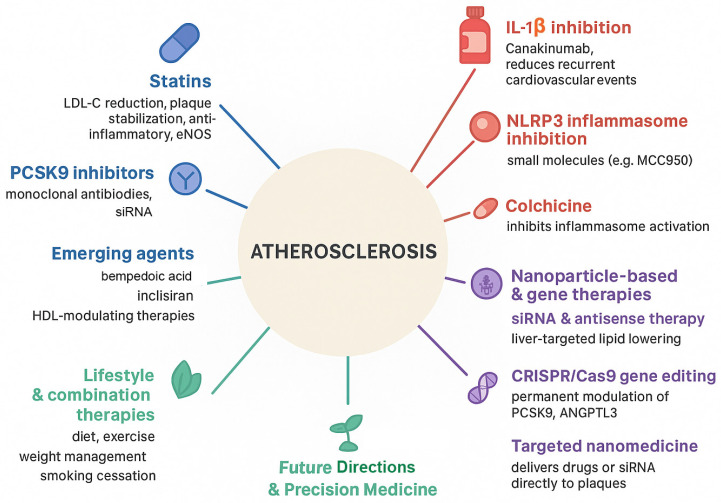
Therapeutic strategies targeting molecular mechanisms of atherosclerosis. This schematic summarizes current, emerging, and prospective therapies aimed at lipid metabolism, inflammation, and vascular remodeling in atherosclerosis. Established lipid-lowering agents, including statins and PCSK9 inhibitors, reduce LDL-cholesterol, enhance plaque stability, and protect endothelial function. Additional therapies, such as bempedoic acid, inclisiran, and HDL-modulating agents, further regulate lipid homeostasis. Anti-inflammatory strategies, including IL-1β inhibitors, NLRP3 inflammasome inhibitors, and colchicine, target innate immune pathways driving plaque progression. Advanced molecular approaches, including nanoparticle-based delivery systems, siRNA and antisense therapies, and CRISPR/Cas9 gene editing, enable precise modulation of key regulators such as PCSK9 and ANGPTL3. Lifestyle interventions remain integral to comprehensive risk reduction. Future directions emphasize precision medicine and targeted nanomedicine to enhance therapeutic specificity and clinical outcomes (Created with BioRender.com).

The recognition that atherosclerosis is a complex interplay of lipid dysregulation, inflammation, and vascular dysfunction has driven the development of increasingly targeted therapies. While traditional lipid-lowering therapies have demonstrated substantial benefit, residual cardiovascular risk remains due to persistent inflammation, immune activation, and plaque vulnerability. This section summarizes current and emerging therapeutic strategies, with an emphasis on molecularly informed approaches (Figure 7).

### 4.1. Lipid-Lowering Therapies

#### 4.1.1. Statins

Statins, inhibitors of HMG-CoA reductase, remain the cornerstone of lipid-lowering therapy [129,130]. Beyond their primary effect in reducing low-density lipoprotein cholesterol (LDL-C), statins exhibit pleiotropic benefits, including enhanced endothelial function, plaque stabilization, attenuation of oxidative stress, and anti-inflammatory activity [131,132]. Mechanistic studies demonstrate that statins upregulate endothelial nitric oxide synthase (eNOS), inhibit nuclear factor kappa B (NF-κB)–mediated transcription of pro-inflammatory genes, and reduce monocyte adhesion to endothelial cells [133,134]. These effects collectively contribute to both early and advanced plaque modulation.

Although statins are one of the widely used therapies to prevent atherosclerotic cardiovascular disease (ASCVD) in patients, statin intolerance affects 5–30% of ASCVD patients, which compromises their efficacy [135,136,137]. Thus, lipid-lowering combination therapy with statins and other reagents, such as Ezetimibe [138], PCSK9 inhibitors [139], and bempedoic acid [140], exhibited improved efficacy for the treatment of ASCVD.

#### 4.1.2. PCSK9 Inhibitors

PCSK9 inhibition has emerged as an effective strategy for lowering LDL cholesterol, particularly in patients with familial hypercholesterolemia or those intolerant to statins. Therapeutic approaches, including monoclonal antibodies and small interfering RNA (siRNA), enhance hepatic LDL receptor recycling, thereby promoting more efficient clearance of circulating LDL-C [141,142]. Beyond their lipid-lowering effects, clinical trials have demonstrated that these agents contribute to a significant reduction in cardiovascular events, highlighting their potential role in comprehensive cardiovascular risk management [141,143].

Especially, Merck’s enlicitide decanoate (formerly MK-0616) is poised to be the first oral PCSK9 inhibitor for lowering LDL-C, targeting patients with hypercholesterolemia [144]. However, PCSK9 inhibitors have limitations that involve their therapeutic resistance for patients with LDLR mutations and immunogenicity concerns [145,146].

#### 4.1.3. Emerging Lipid-Modifying Agents

Novel lipid-lowering drugs, such as bempedoic acid, an ATP citrate lyase inhibitor, complement statin therapy by suppressing hepatic cholesterol synthesis [147]. Inclisiran, a liver-targeted siRNA against PCSK9 mRNA, provides sustained LDL-C reduction with infrequent dosing [148]. Additionally, strategies aimed at enhancing high-density lipoprotein (HDL) functionality and reverse cholesterol transport are under investigation, although translating HDL modulation into tangible clinical benefit remains challenging [149].

### 4.2. Anti-Inflammatory Approaches

#### 4.2.1. IL-1β Inhibition

The Canakinumab Anti-Inflammatory Thrombosis Outcome Study (CANTOS) trial highlighted the causal role of inflammation in atherosclerosis by demonstrating that selective inhibition of interleukin-1β with canakinumab significantly reduced recurrent cardiovascular events independent of lipid levels [8]. The CANTOS trial uses an interleukin-1β monoclonal antibody, canakinumab, which significantly lowered the inflammatory burden but had no impact on low-density lipoprotein (LDL) cholesterol [8,150]. Despite CANTOS supporting the first study targeting inflammation modest cardiovascular benefit in very high-risk patients, the safety concerns and potentially prohibitive cost prevent its widespread application [151].

#### 4.2.2. NLRP3 Inflammasome Inhibition

Given the central role of NLRP3 in macrophage-driven vascular inflammation, small-molecule inhibitors such as MCC950 are under preclinical and early clinical evaluation. These agents disrupt upstream signaling pathways that mediate caspase-1 activation and IL-1β/IL-18 secretion, thereby attenuating inflammatory amplification within plaques [64,152]. Additionally, other NLRP3 inflammasome inhibitors, such as CY-09, Dapansutrile, Tranilast, and Oridonin, have exhibited high efficacy in inhibiting the activity of NLRP3 inflammasome [153].

#### 4.2.3. Gp130 and IL-6 Inhibition

Glycoprotein 130 (gp130) is a signal transducer for the IL-6 cytokine family that modulates atherosclerosis in mice and humans [154]. Low plasma levels of endogenous sgp130 are linked to coronary artery disease in humans. Furthermore, genetic variations in the human IL6ST gene (which encodes gp130) are associated with increased risk of CAD [154]. Gp130 inhibitor, such as sgp130Fc, specifically inhibits IL-6 trans-signaling, which further suppresses inflammation. Treatment with sgp130Fc [155] or anti-IL-6 receptor antibody [156] dramatically reduced atherosclerosis in hypercholesterolemic Ldlr^−/−^ mice. Unlike complete IL-6 blockade, which might have adverse effects, specifically targeting trans-signaling with sgp130-Fc allows for the preservation of “classic” protective or metabolic IL-6 signaling while inhibiting pro-inflammatory effects [157].

#### 4.2.4. Colchicine

Administration of low-dose colchicine inhibits microtubule-dependent activation of the inflammasome and has been associated with a lower incidence of recurrent cardiovascular events in patients following myocardial infarction [158,159]. This effect highlights the potential of targeting immune cell activity more broadly as a therapeutic approach within atherosclerotic plaques.

### 4.3. Nanoparticle-Based and Gene Therapies

#### 4.3.1. siRNA and Antisense Therapies

Nanoparticle-mediated delivery of siRNAs targeting atherogenic genes such as PCSK9 or epsin in hepatocytes demonstrates potent lipid-lowering and anti-atherosclerotic effects in preclinical models [160,161]. Liver-targeted approaches minimize systemic exposure, reducing off-target effects.

#### 4.3.2. CRISPR/Cas9-Based Gene Editing

Emerging gene editing strategies aim to permanently modulate key atherogenic genes, including PCSK9 and ANGPTL3. Preclinical studies in murine and non-human primate models report durable LDL-C reduction and plaque regression with minimal adverse effects [162,163]. VERVE-101 is a CRISPR editing therapy that durably lowers LDL cholesterol in patients with heterozygous familial hypercholesterolemia (HeFH) and atherosclerotic cardiovascular disease (ASCVD) [164]. Additionally, the recent clinical trial of CRISPR-Cas9 gene editing targeting the ANGPTL3 drug, named CTX310, exhibited reduced ANGPTL3 levels [165].

#### 4.3.3. Targeted Nanomedicine

Functionalized nanoparticles carrying ligands for vascular adhesion molecules or macrophage scavenger receptors enable selective delivery of anti-inflammatory agents or siRNAs directly to plaques, enhancing therapeutic efficacy while minimizing systemic toxicity [166,167,168,169,170].

Nanoparticles designed for VCAM-1–mediated gene delivery can selectively accumulate in inflamed endothelial cells [171]. Consequently, suppression of VCAM-1 expression represents a promising and precise anti-inflammatory therapeutic approach in atherosclerosis [172].

In parallel, nanoparticle-based targeting of macrophage scavenger receptors, including CD36, has demonstrated efficacy in atherosclerosis models by attenuating plaque burden, limiting inflammatory responses, and reducing foam cell formation [173]. Moreover, silencing Ca^2+^/calmodulin-dependent protein kinase γ (CaMKIIγ) using siRNA has been shown to ameliorate atherosclerotic lesions in murine models [174]. Collectively, these findings highlight the substantial potential of macrophage-targeted multifunctional nanoparticles for both the detection and prevention of atherosclerotic cardiovascular disease [175].

### 4.4. Lifestyle and Combination Therapies

Lifestyle interventions, such as adherence to a Mediterranean diet, reduced saturated fat intake, regular physical activity, weight management, and smoking cessation, remain essential components of atherosclerosis prevention and management [176,177,178]. When combined with pharmacologic therapies, these measures synergistically improve endothelial function, modulate systemic inflammation, and optimize lipid profiles.

Combination regimens integrating lipid-lowering, anti-inflammatory, and lifestyle interventions demonstrate additive or synergistic benefits in reducing residual cardiovascular risk. Molecularly guided personalization, informed by genomic, proteomic, and metabolomic profiling, represents a promising avenue for precision therapy [36,179].

### 4.5. Future Directions and Precision Medicine

#### 4.5.1. Biomarker-Guided Therapy

Circulating molecular biomarkers, including noncoding RNAs, inflammasome components, and immune cell signatures, hold potential for patient stratification and individualized therapeutic guidance [16,180,181].

#### 4.5.2. Immune Cell Reprogramming

Therapeutic modulation of macrophage polarization, T cell subsets, and smooth muscle phenotypes offers strategies to stabilize plaques or induce regression. Advanced delivery platforms, including nanomedicine and gene therapy, enable targeted modulation of specific cell populations within lesions [71,166,182,183].

#### 4.5.3. Integration of Multi-Omics and Artificial Intelligence

High-throughput analyses across genomics, transcriptomics, proteomics, and metabolomics, integrated with AI-driven modeling, are poised to identify novel molecular targets and enable patient-specific interventions. This approach heralds a new era of precision cardiovascular medicine, wherein therapy is informed by the molecular and cellular profile of both the patient and the lesion [36,179].

## 5. Conclusions and Future Perspectives

Atherosclerosis is a complex, multifactorial disease arising from the intricate interplay between lipid dysregulation, endothelial dysfunction, chronic inflammation, and immune activation. Advances over the past decades have significantly deepened our understanding of its molecular and cellular underpinnings, revealing critical pathways such as endothelial injury and lipoprotein retention, macrophage recruitment and foam cell formation, vascular smooth muscle cell (VSMC) phenotypic switching, extracellular matrix remodeling, and the regulatory roles of emerging molecular mediators, including noncoding RNAs and metabolic reprogramming.

These mechanistic insights have directly informed the development of both established and innovative therapeutic strategies. Conventional lipid-lowering therapies, including statins and PCSK9 inhibitors, remain foundational, effectively reducing LDL-C and modulating plaque biology. Complementing these approaches, anti-inflammatory interventions, such as IL-1β inhibition and low-dose colchicine, underscore the importance of targeting residual cardiovascular risk beyond cholesterol management. Emerging modalities, ranging from nanoparticle-mediated drug delivery and RNA-based therapeutics to CRISPR/Cas9 gene editing, offer precise, cell-specific interventions that minimize systemic toxicity and address previously intractable molecular drivers of atherosclerosis.

Looking forward, the integration of multi-omics analyses, including genomics, transcriptomics, proteomics, and metabolomics, combined with advanced computational tools such as artificial intelligence, promises to refine patient stratification, reveal novel therapeutic targets, and enable truly personalized cardiovascular medicine. Strategies such as biomarker-guided therapy, immune cell reprogramming, and molecularly targeted nanomedicine represent the next frontier in the prevention, stabilization, and regression of atherosclerotic plaques.

In conclusion, integrating insights into the molecular underpinnings of atherosclerosis with novel therapeutic approaches provides a promising avenue for improving disease management. Ongoing investigation into the cellular and molecular processes that govern plaque formation, progression, and destabilization will be critical for translating these findings into clinical interventions and ultimately mitigating the worldwide impact of cardiovascular disease.

## Data Availability

No new data were created or analyzed in this study. Data sharing is not applicable to this article.

## References

[1] Benjamin E.J., Muntner P., Alonso A., Bittencourt M.S., Callaway C.W., Carson A.P., Chamberlain A.M., Chang A.R., Cheng S., Das S.R. (2019). Heart Disease and Stroke Statistics-2019 Update: A Report from the American Heart Association. Circulation.

[2] Roth G.A., Mensah G.A., Fuster V. (2020). The Global Burden of Cardiovascular Diseases and Risks: A Compass for Global Action. J. Am. Coll. Cardiol..

[3] Zhu B., Wu H., Li K.S., Eisa-Beygi S., Singh B., Bielenberg D.R., Huang W., Chen H. (2024). Two sides of the same coin: Non-alcoholic fatty liver disease and atherosclerosis. Vasc. Pharmacol..

[4] Libby P., Buring J.E., Badimon L., Hansson G.K., Deanfield J., Bittencourt M.S., Tokgozoglu L., Lewis E.F. (2019). Atherosclerosis. Nat. Rev. Dis. Primers.

[5] Lusis A.J. (2000). Atherosclerosis. Nature.

[6] Grundy S.M., Cleeman J.I., Daniels S.R., Donato K.A., Eckel R.H., Franklin B.A., Gordon D.J., Krauss R.M., Savage P.J., Smith S.C. (2005). Diagnosis and management of the metabolic syndrome: An American Heart Association/National Heart, Lung, and Blood Institute Scientific Statement. Circulation.

[7] Yusuf S., Hawken S., Ôunpuu S., Dans T., Avezum A., Lanas F., McQueen M., Budaj A., Pais P., Varigos J. (2004). Effect of potentially modifiable risk factors associated with myocardial infarction in 52 countries (the INTERHEART study): Case-control study. Lancet.

[8] Ridker P.M., Everett B.M., Thuren T., MacFadyen J.G., Chang W.H., Ballantyne C., Fonseca F., Nicolau J., Koenig W., Anker S.D. (2017). Antiinflammatory Therapy with Canakinumab for Atherosclerotic Disease. N. Engl. J. Med..

[9] Sabatine M.S., Giugliano R.P., Keech A.C., Honarpour N., Wiviott S.D., Murphy S.A., Kuder J.F., Wang H., Liu T., Wasserman S.M. (2017). Evolocumab and Clinical Outcomes in Patients with Cardiovascular Disease. N. Engl. J. Med..

[10] Libby P., Hansson G.K. (2015). Inflammation and immunity in diseases of the arterial tree: Players and layers. Circ. Res..

[11] Allahverdian S., Chaabane C., Boukais K., Francis G.A., Bochaton-Piallat M.L. (2018). Smooth muscle cell fate and plasticity in atherosclerosis. Cardiovasc. Res..

[12] Williams K.J., Tabas I. (1995). The response-to-retention hypothesis of early atherogenesis. Arterioscler. Thromb. Vasc. Biol..

[13] Tabas I., Garcia-Cardena G., Owens G.K. (2015). Recent insights into the cellular biology of atherosclerosis. J. Cell Biol..

[14] Moore K.J., Sheedy F.J., Fisher E.A. (2013). Macrophages in atherosclerosis: A dynamic balance. Nat. Rev. Immunol..

[15] Bjorkegren J.L.M., Lusis A.J. (2022). Atherosclerosis: Recent developments. Cell.

[16] Feinberg M.W., Moore K.J. (2016). MicroRNA Regulation of Atherosclerosis. Circ. Res..

[17] Back M., Yurdagul A., Tabas I., Oorni K., Kovanen P.T. (2019). Inflammation and its resolution in atherosclerosis: Mediators and therapeutic opportunities. Nat. Rev. Cardiol..

[18] Soumya R.S., Raghu K.G. (2023). Recent advances on nanoparticle-based therapies for cardiovascular diseases. J. Cardiol..

[19] Goldstein J.L., Brown M.S. (2009). The LDL receptor. Arterioscler. Thromb. Vasc. Biol..

[20] Abifadel M., Varret M., Rabes J.P., Allard D., Ouguerram K., Devillers M., Cruaud C., Benjannet S., Wickham L., Erlich D. (2003). Mutations in PCSK9 cause autosomal dominant hypercholesterolemia. Nat. Genet..

[21] Soutar A.K., Naoumova R.P. (2007). Mechanisms of disease: Genetic causes of familial hypercholesterolemia. Nat. Clin. Pract. Cardiovasc. Med..

[22] Tall A.R. (2007). CETP inhibitors to increase HDL cholesterol levels. N. Engl. J. Med..

[23] Rader D.J. (2006). Molecular regulation of HDL metabolism and function: Implications for novel therapies. J. Clin. Investig..

[24] Nordestgaard B.G. (2016). Triglyceride-Rich Lipoproteins and Atherosclerotic Cardiovascular Disease: New Insights From Epidemiology, Genetics, and Biology. Circ. Res..

[25] Libby P. (2002). Inflammation in atherosclerosis. Nature.

[26] Smith A.J., Humphries S.E. (2009). Cytokine and cytokine receptor gene polymorphisms and their functionality. Cytokine Growth Factor Rev..

[27] Szalai C., Duba J., Prohaszka Z., Kalina A., Szabo T., Nagy B., Horvath L., Csaszar A. (2001). Involvement of polymorphisms in the chemokine system in the susceptibility for coronary artery disease (CAD). Coincidence of elevated Lp(a) and MCP-1-2518 G/G genotype in CAD patients. Atherosclerosis.

[28] Cyr A.R., Huckaby L.V., Shiva S.S., Zuckerbraun B.S. (2020). Nitric Oxide and Endothelial Dysfunction. Crit. Care Clin..

[29] Ferguson J.F., Phillips C.M., McMonagle J., Perez-Martinez P., Shaw D.I., Lovegrove J.A., Helal O., Defoort C., Gjelstad I.M., Drevon C.A. (2010). NOS3 gene polymorphisms are associated with risk markers of cardiovascular disease, and interact with omega-3 polyunsaturated fatty acids. Atherosclerosis.

[30] Agerholm-Larsen B., Nordestgaard B.G., Tybjaerg-Hansen A. (2000). ACE gene polymorphism in cardiovascular disease: Meta-analyses of small and large studies in whites. Arterioscler. Thromb. Vasc. Biol..

[31] Kujovich J.L. (2011). Factor V Leiden thrombophilia. Genet. Med..

[32] Junker R., Koch H.G., Auberger K., Munchow N., Ehrenforth S., Nowak-Gottl U. (1999). Prothrombin G20210A gene mutation and further prothrombotic risk factors in childhood thrombophilia. Arterioscler. Thromb. Vasc. Biol..

[33] Khera A.V., Emdin C.A., Drake I., Natarajan P., Bick A.G., Cook N.R., Chasman D.I., Baber U., Mehran R., Rader D.J. (2016). Genetic Risk, Adherence to a Healthy Lifestyle, and Coronary Disease. N. Engl. J. Med..

[34] Kohler H.P., Grant P.J. (2000). Plasminogen-activator inhibitor type 1 and coronary artery disease. N. Engl. J. Med..

[35] Nikpay M., Goel A., Won H.H., Hall L.M., Willenborg C., Kanoni S., Saleheen D., Kyriakou T., Nelson C.P., Hopewell J.C. (2015). A comprehensive 1000 Genomes-based genome-wide association meta-analysis of coronary artery disease. Nat Genet..

[36] Inouye M., Abraham G., Nelson C.P., Wood A.M., Sweeting M.J., Dudbridge F., Lai F.Y., Kaptoge S., Brozynska M., Wang T. (2018). Genomic Risk Prediction of Coronary Artery Disease in 480,000 Adults: Implications for Primary Prevention. J. Am. Coll. Cardiol..

[37] Wierda R.J., Geutskens S.B., Jukema J.W., Quax P.H., van den Elsen P.J. (2010). Epigenetics in atherosclerosis and inflammation. J. Cell. Mol. Med..

[38] O’Sullivan J.W., Raghavan S., Marquez-Luna C., Luzum J.A., Damrauer S.M., Ashley E.A., O’Donnell C.J., Willer C.J., Natarajan P., American Heart Association Council on Genomic and Precision Medicine (2022). Polygenic Risk Scores for Cardiovascular Disease: A Scientific Statement From the American Heart Association. Circulation.

[39] Hamooya B.M., Siame L., Muchaili L., Masenga S.K., Kirabo A. (2025). Metabolic syndrome: Epidemiology, mechanisms, and current therapeutic approaches. Front. Nutr..

[40] Fuertes E., van der Plaat D.A., Minelli C. (2020). Antioxidant genes and susceptibility to air pollution for respiratory and cardiovascular health. Free. Radic. Biol. Med..

[41] Hassan M.G., Elmezain W.A., Baraka D.M., AboElmaaty S.A., Elhassanein A., Ibrahim R.M., Hamed A.A. (2024). Anti-Cancer and Anti-Oxidant Bioactive Metabolites from Aspergillus fumigatus WA7S6 Isolated from Marine Sources: In Vitro and In Silico Studies. Microorganisms.

[42] Grover-Paez F., Zavalza-Gomez A.B. (2009). Endothelial dysfunction and cardiovascular risk factors. Diabetes Res. Clin. Pract..

[43] Davignon J., Ganz P. (2004). Role of endothelial dysfunction in atherosclerosis. Circulation.

[44] Gimbrone M.A., Garcia-Cardena G. (2016). Endothelial Cell Dysfunction and the Pathobiology of Atherosclerosis. Circ. Res..

[45] Tabas I., Williams K.J., Boren J. (2007). Subendothelial lipoprotein retention as the initiating process in atherosclerosis: Update and therapeutic implications. Circulation.

[46] Steinberg D. (2009). The LDL modification hypothesis of atherogenesis: An update. J. Lipid Res..

[47] Levitan I., Volkov S., Subbaiah P.V. (2010). Oxidized LDL: Diversity, patterns of recognition, and pathophysiology. Antioxid. Redox Signal..

[48] Berbee M., Fu Q., Boerma M., Wang J., Kumar K.S., Hauer-Jensen M. (2009). gamma-Tocotrienol ameliorates intestinal radiation injury and reduces vascular oxidative stress after total-body irradiation by an HMG-CoA reductase-dependent mechanism. Radiat. Res..

[49] Moore K.J., Tabas I. (2011). Macrophages in the pathogenesis of atherosclerosis. Cell.

[50] Ley K., Laudanna C., Cybulsky M.I., Nourshargh S. (2007). Getting to the site of inflammation: The leukocyte adhesion cascade updated. Nat. Rev. Immunol..

[51] Hansson G.K. (2005). Inflammation, atherosclerosis, and coronary artery disease. N. Engl. J. Med..

[52] Forstermann U., Xia N., Li H. (2017). Roles of Vascular Oxidative Stress and Nitric Oxide in the Pathogenesis of Atherosclerosis. Circ. Res..

[53] Mestas J., Ley K. (2008). Monocyte-endothelial cell interactions in the development of atherosclerosis. Trends Cardiovasc. Med..

[54] Xu S., Ogura S., Chen J., Little P.J., Moss J., Liu P. (2013). LOX-1 in atherosclerosis: Biological functions and pharmacological modifiers. Cell. Mol. Life Sci..

[55] Han J., Hajjar D.P., Tauras J.M., Nicholson A.C. (1999). Cellular cholesterol regulates expression of the macrophage type B scavenger receptor, CD36. J. Lipid Res..

[56] Mehta J.L., Chen J., Hermonat P.L., Romeo F., Novelli G. (2006). Lectin-like, oxidized low-density lipoprotein receptor-1 (LOX-1): A critical player in the development of atherosclerosis and related disorders. Cardiovasc. Res..

[57] Podrez E.A., Febbraio M., Sheibani N., Schmitt D., Silverstein R.L., Hajjar D.P., Cohen P.A., Frazier W.A., Hoff H.F., Hazen S.L. (2000). Macrophage scavenger receptor CD36 is the major receptor for LDL modified by monocyte-generated reactive nitrogen species. J. Clin. Investig..

[58] Kunjathoor V.V., Febbraio M., Podrez E.A., Moore K.J., Andersson L., Koehn S., Rhee J.S., Silverstein R., Hoff H.F., Freeman M.W. (2002). Scavenger receptors class A-I/II and CD36 are the principal receptors responsible for the uptake of modified low density lipoprotein leading to lipid loading in macrophages. J. Biol. Chem..

[59] Moore K.J., Koplev S., Fisher E.A., Tabas I., Bjorkegren J.L.M., Doran A.C., Kovacic J.C. (2018). Macrophage Trafficking, Inflammatory Resolution, and Genomics in Atherosclerosis: JACC Macrophage in CVD Series (Part 2). J. Am. Coll. Cardiol..

[60] Xiang P., Blanchard V., Francis G.A. (2022). Smooth Muscle Cell-Macrophage Interactions Leading to Foam Cell Formation in Atherosclerosis: Location, Location, Location. Front. Physiol..

[61] Tabas I. (2005). Consequences and therapeutic implications of macrophage apoptosis in atherosclerosis: The importance of lesion stage and phagocytic efficiency. Arterioscler. Thromb. Vasc. Biol..

[62] Kojima Y., Weissman I.L., Leeper N.J. (2017). The Role of Efferocytosis in Atherosclerosis. Circulation.

[63] Tabas I., Lichtman A.H. (2017). Monocyte-Macrophages and T Cells in Atherosclerosis. Immunity.

[64] Duewell P., Kono H., Rayner K.J., Sirois C.M., Vladimer G., Bauernfeind F.G., Abela G.S., Franchi L., Nunez G., Schnurr M. (2010). NLRP3 inflammasomes are required for atherogenesis and activated by cholesterol crystals. Nature.

[65] Rajamaki K., Lappalainen J., Oorni K., Valimaki E., Matikainen S., Kovanen P.T., Eklund K.K. (2010). Cholesterol crystals activate the NLRP3 inflammasome in human macrophages: A novel link between cholesterol metabolism and inflammation. PLoS ONE.

[66] Sheedy F.J., Grebe A., Rayner K.J., Kalantari P., Ramkhelawon B., Carpenter S.B., Becker C.E., Ediriweera H.N., Mullick A.E., Golenbock D.T. (2013). CD36 coordinates NLRP3 inflammasome activation by facilitating intracellular nucleation of soluble ligands into particulate ligands in sterile inflammation. Nat. Immunol..

[67] Cochain C., Vafadarnejad E., Arampatzi P., Pelisek J., Winkels H., Ley K., Wolf D., Saliba A.E., Zernecke A. (2018). Single-Cell RNA-Seq Reveals the Transcriptional Landscape and Heterogeneity of Aortic Macrophages in Murine Atherosclerosis. Circ. Res..

[68] Winkels H., Ehinger E., Vassallo M., Buscher K., Dinh H.Q., Kobiyama K., Hamers A.A.J., Cochain C., Vafadarnejad E., Saliba A.-E. (2018). Atlas of the Immune Cell Repertoire in Mouse Atherosclerosis Defined by Single-Cell RNA-Sequencing and Mass Cytometry. Circ. Res..

[69] Lin J.D., Nishi H., Poles J., Niu X., McCauley C., Rahman K., Brown E.J., Yeung S.T., Vozhilla N., Weinstock A. (2019). Single-cell analysis of fate-mapped macrophages reveals heterogeneity, including stem-like properties, during atherosclerosis progression and regression. JCI Insight.

[70] Chinetti-Gbaguidi G., Daoudi M., Rosa M., Vinod M., Louvet L., Copin C., Fanchon M., Vanhoutte J., Derudas B., Belloy L. (2017). Human Alternative Macrophages Populate Calcified Areas of Atherosclerotic Lesions and Display Impaired RANKL-Induced Osteoclastic Bone Resorption Activity. Circ. Res..

[71] Tabas I., Bornfeldt K.E. (2016). Macrophage Phenotype and Function in Different Stages of Atherosclerosis. Circ. Res..

[72] Galis Z.S., Sukhova G.K., Lark M.W., Libby P. (1994). Increased expression of matrix metalloproteinases and matrix degrading activity in vulnerable regions of human atherosclerotic plaques. J. Clin. Investig..

[73] Libby P. (2021). The changing landscape of atherosclerosis. Nature.

[74] Newby A.C. (2007). Metalloproteinases and vulnerable atherosclerotic plaques. Trends Cardiovasc. Med..

[75] Bennett M.R., Sinha S., Owens G.K. (2016). Vascular Smooth Muscle Cells in Atherosclerosis. Circ. Res..

[76] Owens G.K., Kumar M.S., Wamhoff B.R. (2004). Molecular regulation of vascular smooth muscle cell differentiation in development and disease. Physiol. Rev..

[77] Basatemur G.L., Jorgensen H.F., Clarke M.C.H., Bennett M.R., Mallat Z. (2019). Vascular smooth muscle cells in atherosclerosis. Nat. Rev. Cardiol..

[78] Allahverdian S., Chehroudi A.C., McManus B.M., Abraham T., Francis G.A. (2014). Contribution of intimal smooth muscle cells to cholesterol accumulation and macrophage-like cells in human atherosclerosis. Circulation.

[79] Virmani R., Kolodgie F.D., Burke A.P., Farb A., Schwartz S.M. (2000). Lessons from sudden coronary death: A comprehensive morphological classification scheme for atherosclerotic lesions. Arterioscler. Thromb. Vasc. Biol..

[80] Clarke M.C., Figg N., Maguire J.J., Davenport A.P., Goddard M., Littlewood T.D., Bennett M.R. (2006). Apoptosis of vascular smooth muscle cells induces features of plaque vulnerability in atherosclerosis. Nat. Med..

[81] Bekkering S., Quintin J., Joosten L.A., van der Meer J.W., Netea M.G., Riksen N.P. (2014). Oxidized low-density lipoprotein induces long-term proinflammatory cytokine production and foam cell formation via epigenetic reprogramming of monocytes. Arterioscler. Thromb. Vasc. Biol..

[82] Zaina S., Heyn H., Carmona F.J., Varol N., Sayols S., Condom E., Ramirez-Ruz J., Gomez A., Goncalves I., Moran S. (2014). DNA methylation map of human atherosclerosis. Circ. Cardiovasc. Genet..

[83] Movassagh M., Choy M.K., Knowles D.A., Cordeddu L., Haider S., Down T., Siggens L., Vujic A., Simeoni I., Penkett C. (2011). Distinct epigenomic features in end-stage failing human hearts. Circulation.

[84] Ouimet M., Ediriweera H.N., Gundra U.M., Sheedy F.J., Ramkhelawon B., Hutchison S.B., Rinehold K., van Solingen C., Fullerton M.D., Cecchini K. (2015). MicroRNA-33-dependent regulation of macrophage metabolism directs immune cell polarization in atherosclerosis. J. Clin. Investig..

[85] Wang B., Zhu X., Kim Y., Li J., Huang S., Saleem S., Li R.-C., Xu Y., Dore S., Cao W. (2012). Histone deacetylase inhibition activates transcription factor Nrf2 and protects against cerebral ischemic damage. Free Radic. Biol. Med..

[86] Hamidi T., Singh A.K., Veland N., Vemulapalli V., Chen J., Hardikar S., Bao J., Fry C.J., Yang V., Lee K.A. (2018). Identification of Rpl29 as a major substrate of the lysine methyltransferase Set7/9. J. Biol. Chem..

[87] Ouimet M., Ediriweera H., Afonso M.S., Ramkhelawon B., Singaravelu R., Liao X., Bandler R.C., Rahman K., Fisher E.A., Rayner K.J. (2017). microRNA-33 Regulates Macrophage Autophagy in Atherosclerosis. Arterioscler. Thromb. Vasc. Biol..

[88] Holdt L.M., Beutner F., Scholz M., Gielen S., Gabel G., Bergert H., Schuler G., Thiery J., Teupser D. (2010). ANRIL expression is associated with atherosclerosis risk at chromosome 9p21. Arterioscler. Thromb. Vasc. Biol..

[89] Zernecke A., Bidzhekov K., Noels H., Shagdarsuren E., Gan L., Denecke B., Hristov M., Koppel T., Jahantigh M.N., Lutgens E. (2009). Delivery of microRNA-126 by apoptotic bodies induces CXCL12-dependent vascular protection. Sci. Signal..

[90] Bonauer A., Carmona G., Iwasaki M., Mione M., Koyanagi M., Fischer A., Burchfield J., Fox H., Doebele C., Ohtani K. (2009). MicroRNA-92a controls angiogenesis and functional recovery of ischemic tissues in mice. Science.

[91] Kumarswamy R., Volkmann I., Thum T. (2011). Regulation and function of miRNA-21 in health and disease. RNA Biol..

[92] Rayner K.J., Suarez Y., Davalos A., Parathath S., Fitzgerald M.L., Tamehiro N., Fisher E.A., Moore K.J., Fernandez-Hernando C. (2010). MiR-33 contributes to the regulation of cholesterol homeostasis. Science.

[93] Esau C., Davis S., Murray S.F., Yu X.X., Pandey S.K., Pear M., Watts L., Booten S.L., Graham M., McKay R. (2006). miR-122 regulation of lipid metabolism revealed by in vivo antisense targeting. Cell Metab..

[94] Wagschal A., Najafi-Shoushtari S.H., Wang L., Goedeke L., Sinha S., deLemos A.S., Black J.C., Ramirez C.M., Li Y., Tewhey R. (2015). Genome-wide identification of microRNAs regulating cholesterol and triglyceride homeostasis. Nat. Med..

[95] O’Connell R.M., Taganov K.D., Boldin M.P., Cheng G., Baltimore D. (2007). MicroRNA-155 is induced during the macrophage inflammatory response. Proc. Natl. Acad. Sci. USA.

[96] Taganov K.D., Boldin M.P., Chang K.J., Baltimore D. (2006). NF-kappaB-dependent induction of microRNA miR-146, an inhibitor targeted to signaling proteins of innate immune responses. Proc. Natl. Acad. Sci. USA.

[97] Qin Z., Wang P.Y., Su D.F., Liu X. (2016). miRNA-124 in Immune System and Immune Disorders. Front. Immunol..

[98] Cordes K.R., Sheehy N.T., White M.P., Berry E.C., Morton S.U., Muth A.N., Lee T.H., Miano J.M., Ivey K.N., Srivastava D. (2009). miR-145 and miR-143 regulate smooth muscle cell fate and plasticity. Nature.

[99] Liu X., Cheng Y., Zhang S., Lin Y., Yang J., Zhang C. (2009). A necessary role of miR-221 and miR-222 in vascular smooth muscle cell proliferation and neointimal hyperplasia. Circ. Res..

[100] Boon R.A., Seeger T., Heydt S., Fischer A., Hergenreider E., Horrevoets A.J., Vinciguerra M., Rosenthal N., Sciacca S., Pilato M. (2011). MicroRNA-29 in aortic dilation: Implications for aneurysm formation. Circ. Res..

[101] Sallam T., Jones M.C., Gilliland T., Zhang L., Wu X., Eskin A., Sandhu J., Casero D., Vallim T.Q.d.A., Hong C. (2016). Feedback modulation of cholesterol metabolism by the lipid-responsive non-coding RNA LeXis. Nature.

[102] Sallam T., Jones M., Thomas B.J., Wu X., Gilliland T., Qian K., Eskin A., Casero D., Zhang Z., Sandhu J. (2018). Transcriptional regulation of macrophage cholesterol efflux and atherogenesis by a long noncoding RNA. Nat. Med..

[103] Michalik K.M., You X., Manavski Y., Doddaballapur A., Zornig M., Braun T., John D., Ponomareva Y., Chen W., Uchida S. (2014). Long noncoding RNA MALAT1 regulates endothelial cell function and vessel growth. Circ. Res..

[104] Chen F., Chen J., Yang L., Liu J., Zhang X., Zhang Y., Tu Q., Yin D., Lin D., Wong P.P. (2019). Extracellular vesicle-packaged HIF-1alpha-stabilizing lncRNA from tumour-associated macrophages regulates aerobic glycolysis of breast cancer cells. Nat. Cell Biol..

[105] Carpenter S., Aiello D., Atianand M.K., Ricci E.P., Gandhi P., Hall L.L., Byron M., Monks B., Henry-Bezy M., Lawrence J.B. (2013). A long noncoding RNA mediates both activation and repression of immune response genes. Science.

[106] Song Y., Wang T., Mu C., Gui W., Deng Y., Ma R. (2022). LncRNA SENCR overexpression attenuated the proliferation, migration and phenotypic switching of vascular smooth muscle cells in aortic dissection via the miR-206/myocardin axis. Nutr. Metab. Cardiovasc. Dis..

[107] Ballantyne M.D., Pinel K., Dakin R., Vesey A.T., Diver L., Mackenzie R., Garcia R., Welsh P., Sattar N., Hamilton G. (2016). Smooth Muscle Enriched Long Noncoding RNA (SMILR) Regulates Cell Proliferation. Circulation.

[108] Yan B., Yao J., Liu J.Y., Li X.M., Wang X.Q., Li Y.J., Tao Z.F., Song Y.C., Chen Q., Jiang Q. (2015). lncRNA-MIAT regulates microvascular dysfunction by functioning as a competing endogenous RNA. Circ. Res..

[109] Yoon J.H., Abdelmohsen K., Srikantan S., Yang X., Martindale J.L., De S., Huarte M., Zhan M., Becker K.G., Gorospe M. (2012). LincRNA-p21 suppresses target mRNA translation. Mol. Cell.

[110] Hennessy E.J., van Solingen C., Scacalossi K.R., Ouimet M., Afonso M.S., Prins J., Koelwyn G.J., Sharma M., Ramkhelawon B., Carpenter S. (2019). The long noncoding RNA CHROME regulates cholesterol homeostasis in primate. Nat. Metab..

[111] Schulte C., Molz S., Appelbaum S., Karakas M., Ojeda F., Lau D.M., Hartmann T., Lackner K.J., Westermann D., Schnabel R.B. (2015). miRNA-197 and miRNA-223 Predict Cardiovascular Death in a Cohort of Patients with Symptomatic Coronary Artery Disease. PLoS ONE.

[112] Anastasiadou E., Jacob L.S., Slack F.J. (2018). Non-coding RNA networks in cancer. Nat. Rev. Cancer.

[113] van Rooij E., Olson E.N. (2012). MicroRNA therapeutics for cardiovascular disease: Opportunities and obstacles. Nat. Rev. Drug Discov..

[114] Bekkering S., Arts R.J.W., Novakovic B., Kourtzelis I., van der Heijden C., Li Y., Popa C.D., Ter Horst R., van Tuijl J., Netea-Maier R.T. (2018). Metabolic Induction of Trained Immunity through the Mevalonate Pathway. Cell.

[115] Chen B.H., Marioni R.E., Colicino E., Peters M.J., Ward-Caviness C.K., Tsai P.C., Roetker N.S., Just A.C., Demerath E.W., Guan W. (2016). DNA methylation-based measures of biological age: Meta-analysis predicting time to death. Aging.

[116] Zhang J.A., Mortazavi A., Williams B.A., Wold B.J., Rothenberg E.V. (2012). Dynamic transformations of genome-wide epigenetic marking and transcriptional control establish T cell identity. Cell.

[117] Kelly B., O’Neill L.A. (2015). Metabolic reprogramming in macrophages and dendritic cells in innate immunity. Cell Res..

[118] O’Neill L.A., Kishton R.J., Rathmell J. (2016). A guide to immunometabolism for immunologists. Nat. Rev. Immunol..

[119] Palsson-McDermott E.M., Curtis A.M., Goel G., Lauterbach M.A.R., Sheedy F.J., Gleeson L.E., van den Bosch M.W.M., Quinn S.R., Domingo-Fernandez R., Johnston D.G.W. (2015). Pyruvate Kinase M2 Regulates Hif-1alpha Activity and IL-1beta Induction and Is a Critical Determinant of the Warburg Effect in LPS-Activated Macrophages. Cell Metab..

[120] Kim S.Y., Nair M.G. (2019). Macrophages in wound healing: Activation and plasticity. Immunol. Cell Biol..

[121] Tall A.R., Yvan-Charvet L. (2015). Cholesterol, inflammation and innate immunity. Nat. Rev. Immunol..

[122] Westerterp M., Murphy A.J., Wang M., Pagler T.A., Vengrenyuk Y., Kappus M.S., Gorman D.J., Nagareddy P.R., Zhu X., Abramowicz S. (2013). Deficiency of ATP-binding cassette transporters A1 and G1 in macrophages increases inflammation and accelerates atherosclerosis in mice. Circ. Res..

[123] Wang N., Tall A.R. (2003). Regulation and mechanisms of ATP-binding cassette transporter A1-mediated cellular cholesterol efflux. Arterioscler. Thromb. Vasc. Biol..

[124] Yvan-Charvet L., Pagler T.A., Seimon T.A., Thorp E., Welch C.L., Witztum J.L., Tabas I., Tall A.R. (2010). ABCA1 and ABCG1 protect against oxidative stress-induced macrophage apoptosis during efferocytosis. Circ. Res..

[125] Ouimet M., Hennessy E.J., van Solingen C., Koelwyn G.J., Hussein M.A., Ramkhelawon B., Rayner K.J., Temel R.E., Perisic L., Hedin U. (2016). miRNA Targeting of Oxysterol-Binding Protein-Like 6 Regulates Cholesterol Trafficking and Efflux. Arterioscler. Thromb. Vasc. Biol..

[126] Citrin K.M., Fernandez-Hernando C., Suarez Y. (2021). MicroRNA regulation of cholesterol metabolism. Ann. N. Y. Acad. Sci..

[127] Saxton R.A., Sabatini D.M. (2017). mTOR Signaling in Growth, Metabolism, and Disease. Cell.

[128] Sutherland T.E., Dyer D.P., Allen J.E. (2023). The extracellular matrix and the immune system: A mutually dependent relationship. Science.

[129] Baigent C., Blackwell L., Emberson J., Holland L.E., Reith C., Bhala N., Peto R., Barnes E.H., Keech A., Cholesterol Treatment Trialists’ (CTT) Collaboration (2010). Efficacy and safety of more intensive lowering of LDL cholesterol: A meta-analysis of data from 170,000 participants in 26 randomised trials. Lancet.

[130] Ridker P.M., Cook N.R., Lee I.M., Gordon D., Gaziano J.M., Manson J.E., Hennekens C.H., Buring J.E. (2005). A randomized trial of low-dose aspirin in the primary prevention of cardiovascular disease in women. N. Engl. J. Med..

[131] Liao J.K. (2002). Beyond lipid lowering: The role of statins in vascular protection. Int. J. Cardiol..

[132] Davignon J. (2004). Beneficial cardiovascular pleiotropic effects of statins. Circulation.

[133] Spieker L.E., Hurlimann D., Ruschitzka F., Corti R., Enseleit F., Shaw S., Hayoz D., Deanfield J.E., Luscher T.F., Noll G. (2002). Mental stress induces prolonged endothelial dysfunction via endothelin-A receptors. Circulation.

[134] Kwak B., Mulhaupt F., Myit S., Mach F. (2000). Statins as a newly recognized type of immunomodulator. Nat. Med..

[135] Cheeley M.K., Saseen J.J., Agarwala A., Ravilla S., Ciffone N., Jacobson T.A., Dixon D.L., Maki K.C. (2022). NLA scientific statement on statin intolerance: A new definition and key considerations for ASCVD risk reduction in the statin intolerant patient. J. Clin. Lipidol..

[136] Fitchett D.H., Hegele R.A., Verma S. (2015). Cardiology patient page. Statin Intolerance Circ..

[137] Mesi O., Lin C., Ahmed H., Cho L.S. (2021). Statin intolerance and new lipid-lowering treatments. Clevel. Clin. J. Med..

[138] Banach M., Jaiswal V., Ang S.P., Sawhney A., Deb N., Amarenco P., Gaita D., Reiner Z., Pecin I., Lavie C.J. (2025). Impact of Lipid-Lowering Combination Therapy With Statins and Ezetimibe vs Statin Monotherapy on the Reduction of Cardiovascular Outcomes: A Meta-analysis. Mayo Clin. Proc..

[139] Gallego-Colon E., Daum A., Yosefy C. (2020). Statins and PCSK9 inhibitors: A new lipid-lowering therapy. Eur. J. Pharmacol..

[140] Banach M., Duell P.B., Gotto A.M., Laufs U., Leiter L.A., Mancini G.B.J., Ray K.K., Flaim J., Ye Z., Catapano A.L. (2020). Association of Bempedoic Acid Administration With Atherogenic Lipid Levels in Phase 3 Randomized Clinical Trials of Patients With Hypercholesterolemia. JAMA Cardiol..

[141] Sabatine M.S., Giugliano R.P., Wiviott S.D., Raal F.J., Blom D.J., Robinson J., Ballantyne C.M., Somaratne R., Legg J., Wasserman S.M. (2015). Efficacy and safety of evolocumab in reducing lipids and cardiovascular events. N. Engl. J. Med..

[142] Ray K.K., Landmesser U., Leiter L.A., Kallend D., Dufour R., Karakas M., Hall T., Troquay R.P., Turner T., Visseren F.L. (2017). Inclisiran in Patients at High Cardiovascular Risk with Elevated LDL Cholesterol. N. Engl. J. Med..

[143] Robinson J.G., Farnier M., Krempf M., Bergeron J., Luc G., Averna M., Stroes E.S., Langslet G., Raal F.J., El Shahawy M. (2015). Efficacy and safety of alirocumab in reducing lipids and cardiovascular events. N. Engl. J. Med..

[144] Ballantyne C.M., Gellis L., Tardif J.C., Banka P., Navar A.M., Asprusten E.A., Scott R., Stroes E.S.G., Froman S., Mendizabal G. (2025). Efficacy and Safety of Oral PCSK9 Inhibitor Enlicitide in Adults With Heterozygous Familial Hypercholesterolemia: A Randomized Clinical Trial. JAMA.

[145] Zhang Z., Yang R., Zhu J., Yang X., Luo H., Wang H., Luo X. (2024). Failure of lipid control by PCSK9 inhibitors in compound heterozygous familial hypercholesterolemia complicated with premature myocardial infarction: A case report. Clin. Case Rep..

[146] Guan Y., Liu X., Yang Z., Zhu X., Liu M., Du M., Pan X., Wang Y. (2025). PCSK9 Promotes LDLR Degradation by Preventing SNX17-Mediated LDLR Recycling. Circulation.

[147] Ray K.K., Bays H.E., Catapano A.L., Lalwani N.D., Bloedon L.T., Sterling L.R., Robinson P.L., Ballantyne C.M., CLEAR Harmony Trial (2019). Safety and Efficacy of Bempedoic Acid to Reduce LDL Cholesterol. N. Engl. J. Med..

[148] Ray K.K., Wright R.S., Kallend D., Koenig W., Leiter L.A., Raal F.J., Bisch J.A., Richardson T., Jaros M., Wijngaard P.L.J. (2020). Two Phase 3 Trials of Inclisiran in Patients with Elevated LDL Cholesterol. N. Engl. J. Med..

[149] Rader D.J., Hovingh G.K. (2014). HDL and cardiovascular disease. Lancet.

[150] Everett B.M., MacFadyen J.G., Thuren T., Libby P., Glynn R.J., Ridker P.M. (2020). Inhibition of Interleukin-1beta and Reduction in Atherothrombotic Cardiovascular Events in the CANTOS Trial. J. Am. Coll. Cardiol..

[151] Baylis R.A., Gomez D., Mallat Z., Pasterkamp G., Owens G.K. (2017). The CANTOS Trial: One Important Step for Clinical Cardiology but a Giant Leap for Vascular Biology. Arterioscler. Thromb. Vasc. Biol..

[152] Coll R.C., Robertson A.A., Chae J.J., Higgins S.C., Munoz-Planillo R., Inserra M.C., Vetter I., Dungan L.S., Monks B.G., Stutz A. (2015). A small-molecule inhibitor of the NLRP3 inflammasome for the treatment of inflammatory diseases. Nat. Med..

[153] Tanase D.M., Valasciuc E., Gosav E.M., Ouatu A., Buliga-Finis O.N., Floria M., Maranduca M.A., Serban I.L. (2023). Portrayal of NLRP3 Inflammasome in Atherosclerosis: Current Knowledge and Therapeutic Targets. Int. J. Mol. Sci..

[154] Luchtefeld M., Schunkert H., Stoll M., Selle T., Lorier R., Grote K., Sagebiel C., Jagavelu K., Tietge U.J., Assmus U. (2007). Signal transducer of inflammation gp130 modulates atherosclerosis in mice and man. J. Exp. Med..

[155] Schuett H., Oestreich R., Waetzig G.H., Annema W., Luchtefeld M., Hillmer A., Bavendiek U., von Felden J., Divchev D., Kempf T. (2012). Transsignaling of interleukin-6 crucially contributes to atherosclerosis in mice. Arterioscler. Thromb. Vasc. Biol..

[156] Ridker P.M., Rane M. (2021). Interleukin-6 Signaling and Anti-Interleukin-6 Therapeutics in Cardiovascular Disease. Circ. Res..

[157] Rose-John S., Jenkins B.J., Garbers C., Moll J.M., Scheller J. (2023). Targeting IL-6 trans-signalling: Past, present and future prospects. Nat. Rev. Immunol..

[158] Tardif J.C., Kouz S., Waters D.D., Bertrand O.F., Diaz R., Maggioni A.P., Pinto F.J., Ibrahim R., Gamra H., Kiwan G.S. (2019). Efficacy and Safety of Low-Dose Colchicine after Myocardial Infarction. N. Engl. J. Med..

[159] Nidorf S.M., Fiolet A.T.L., Mosterd A., Eikelboom J.W., Schut A., Opstal T.S.J., The S.H.K., Xu X.F., Ireland M.A., Lenderink T. (2020). Colchicine in Patients with Chronic Coronary Disease. N. Engl. J. Med..

[160] Kaczmarek J.C., Kowalski P.S., Anderson D.G. (2017). Advances in the delivery of RNA therapeutics: From concept to clinical reality. Genome Med..

[161] Zhu B., Gupta K., Cui K., Wang B., Singh B., Gao J., Linton M., Chen K., Chen H. (2025). Targeting Liver Epsins Ameliorates Dyslipidemia in Atherosclerosis through Inhibition of Proprotein Convertase Subtilisin/Kexin Type 9-Mediated Low-density Lipoprotein Receptor Degradation. Circulation.

[162] Musunuru K., Chadwick A.C., Mizoguchi T., Garcia S.P., DeNizio J.E., Reiss C.W., Wang K., Iyer S., Dutta C., Clendaniel V. (2021). In vivo CRISPR base editing of PCSK9 durably lowers cholesterol in primates. Nature.

[163] Chadwick A.C., Evitt N.H., Lv W., Musunuru K. (2018). Reduced Blood Lipid Levels With In Vivo CRISPR-Cas9 Base Editing of ANGPTL3. Circulation.

[164] Horie T., Ono K. (2024). VERVE-101: A promising CRISPR-based gene editing therapy that reduces LDL-C and PCSK9 levels in HeFH patients. Eur. Heart J.-Cardiovasc. Pharmacother..

[165] Laffin L.J., Nicholls S.J., Scott R.S., Clifton P.M., Baker J., Sarraju A., Singh S., Wang Q., Wolski K., Xu H. (2025). Phase 1 Trial of CRISPR-Cas9 Gene Editing Targeting ANGPTL3. N. Engl. J. Med..

[166] Cheng Q., Wei T., Farbiak L., Johnson L.T., Dilliard S.A., Siegwart D.J. (2020). Selective organ targeting (SORT) nanoparticles for tissue-specific mRNA delivery and CRISPR-Cas gene editing. Nat. Nanotechnol..

[167] Zhang C.Y., Lin W., Gao J., Shi X., Davaritouchaee M., Nielsen A.E., Mancini R.J., Wang Z. (2019). pH-Responsive Nanoparticles Targeted to Lungs for Improved Therapy of Acute Lung Inflammation/Injury. ACS Appl. Mater. Interfaces.

[168] Chen W., Schilperoort M., Cao Y., Shi J., Tabas I., Tao W. (2022). Macrophage-targeted nanomedicine for the diagnosis and treatment of atherosclerosis. Nat. Rev. Cardiol..

[169] Hossaini Nasr S., Huang X. (2021). Nanotechnology for Targeted Therapy of Atherosclerosis. Front. Pharmacol..

[170] Luo Y., Guo Y., Wang H., Yu M., Hong K., Li D., Li R., Wen B., Hu D., Chang L. (2021). Phospholipid nanoparticles: Therapeutic potentials against atherosclerosis via reducing cholesterol crystals and inhibiting inflammation. eBioMedicine.

[171] Yang H., Zhao F., Li Y., Xu M., Li L., Wu C., Miyoshi H., Liu Y. (2013). VCAM-1-targeted core/shell nanoparticles for selective adhesion and delivery to endothelial cells with lipopolysaccharide-induced inflammation under shear flow and cellular magnetic resonance imaging in vitro. Int. J. Nanomed..

[172] Pickett J.R., Wu Y., Zacchi L.F., Ta H.T. (2023). Targeting endothelial vascular cell adhesion molecule-1 in atherosclerosis: Drug discovery and development of vascular cell adhesion molecule-1-directed novel therapeutics. Cardiovasc. Res..

[173] Lipinski M.J., Frias J.C., Amirbekian V., Briley-Saebo K.C., Mani V., Samber D., Abbate A., Aguinaldo J.G.S., Massey D., Fuster V. (2009). Macrophage-specific lipid-based nanoparticles improve cardiac magnetic resonance detection and characterization of human atherosclerosis. JACC: Cardiovasc. Imaging.

[174] Tao W., Yurdagul A., Kong N., Li W., Wang X., Doran A.C., Feng C., Wang J., Islam M.A., Farokhzad O.C. (2020). siRNA nanoparticles targeting CaMKIIgamma in lesional macrophages improve atherosclerotic plaque stability in mice. Sci. Transl. Med..

[175] Nankivell V., Vidanapathirana A.K., Hoogendoorn A., Tan J.T.M., Verjans J., Psaltis P.J., Hutchinson M.R., Gibson B.C., Lu Y., Goldys E. (2024). Targeting macrophages with multifunctional nanoparticles to detect and prevent atherosclerotic cardiovascular disease. Cardiovasc. Res..

[176] Estruch R., Ros E., Salas-Salvado J., Covas M.I., Corella D., Aros F., Gomez-Gracia E., Ruiz-Gutierrez V., Fiol M., Lapetra J. (2018). Primary Prevention of Cardiovascular Disease with a Mediterranean Diet Supplemented with Extra-Virgin Olive Oil or Nuts. N. Engl. J. Med..

[177] Mozaffarian D., Hao T., Rimm E.B., Willett W.C., Hu F.B. (2011). Changes in diet and lifestyle and long-term weight gain in women and men. N. Engl. J. Med..

[178] Piepoli M.F., Hoes A.W., Agewall S., Albus C., Brotons C., Catapano A.L., Cooney M.T., Corra U., Cosyns B., Deaton C. (2016). 2016 European Guidelines on cardiovascular disease prevention in clinical practice: The Sixth Joint Task Force of the European Society of Cardiology and Other Societies on Cardiovascular Disease Prevention in Clinical Practice (constituted by representatives of 10 societies and by invited experts)Developed with the special contribution of the European Association for Cardiovascular Prevention & Rehabilitation (EACPR). Eur. Heart J..

[179] Topol E.J. (2019). High-performance medicine: The convergence of human and artificial intelligence. Nat. Med..

[180] Ridker P.M., Luscher T.F. (2014). Anti-inflammatory therapies for cardiovascular disease. Eur. Heart J..

[181] Doring N., Kramer N., Mikhailova V., Brand M., Kruger T.H.C., Vowe G. (2021). Sexual Interaction in Digital Contexts and Its Implications for Sexual Health: A Conceptual Analysis. Front. Psychol..

[182] Chinetti-Gbaguidi G., Colin S., Staels B. (2015). Macrophage subsets in atherosclerosis. Nat. Rev. Cardiol..

[183] Li X., Qi H., Cui W., Wang Z., Fu X., Li T., Ma H., Yang Y., Yu T. (2022). Recent advances in targeted delivery of non-coding RNA-based therapeutics for atherosclerosis. Mol. Ther..

